# Transcriptomic Insights into the Antifungal Effects of Magnolol on the Growth and Mycotoxin Production of *Alternaria alternata*

**DOI:** 10.3390/toxins12100665

**Published:** 2020-10-20

**Authors:** Liuqing Wang, Duo Wang, Shuzhi Yuan, Xiaoyuan Feng, Meng Wang

**Affiliations:** 1 Beijing Research Center for Agricultural Standards and Testing, Beijing Academy of Agriculture and Forestry Sciences, Haidian District, Beijing 100097, China; wanglq@brcast.org.cn (L.W.); wangduo@brcast.org.cn (D.W.); fengxy@brcast.org.cn (X.F.); 2 Laboratory of Quality & Safety Risk Assessment for Agro-Products (Beijing), Ministry of Agriculture and Rural Affairs, Haidian District, Beijing 100097, China; 3 Beijing Vegetable Research Center, Beijing Academy of Agriculture and Forestry Sciences, Haidian District, Beijing 100097, China; yuanshuzhi@nercv.org

**Keywords:** *Alternaria alternata*, mycotoxin, alternariol, magnolol, transcriptome

## Abstract

*Alternaria alternata* is an important phytopathogen causing fruit black rot and also producing a variety of mycotoxins, such as alternariol (AOH) and alternariol monomethyl ether (AME) as two main contaminants. This could lead to economic losses of agricultural products as well as human health risks. In this study, magnolol extracted from the traditional Chinese herb, *Mangnolia officinalis*, exhibited an obvious antifungal property and could completely suppress the mycelial growth at 100 μM. Morphological differences of *A. alternata* were observed to be significantly shrunk and wrinkled after the exposure to magnolol. Furthermore, AOH and AME were no longer produced in response to 50 μM of magnolol. To uncover the antifungal and antimycotoxigenic mechanisms, the transcriptomic profiles of *A. alternata*—treated with or without magnolol—were evaluated. The clustered genes responsible for AOH and AME biosynthesis were obviously less transcribed under magnolol stress and this was further confirmed by qRT-PCR. The global regulators of carbon and nitrogen utilization, such as CreA and NmrA, were significantly down-regulated and this possibly caused the reduction in mycotoxins. In addition, fatty acid β-oxidation was regarded to contribute to polyketide mycotoxin production for the supply of precursor acetyl-CoA while the expression of these related genes was inhibited. The response to magnolol led to the marked alteration of oxidative stress and the down-expression of the mitogen-activated protein kinase (MAPK) signaling pathway from the transcriptome data and the determination of peroxidase (POD), superoxide dismutase (SOD) and glutathione (GSH) assays. This above might be the very reason for the growth supression and mycotoxin production of *A. alternata* by magnolol. This study provides new insights into its potential as an important active ingredient for the control of *A. alternata* and its mycotoxins in fruits and their products.

## 1. Introduction

The genus of *Alternaria* is one of the most important postharvest phytopathogens as the causal agent of fruit black rot, resulting in agricultural yield losses worldwide. *A. alternata* is the most common species among the genus *Alternaria* containing a variety of host-specific pathogenic strains [1]. In addition to economic losses and fruit quality reduction, they can produce multiple mycotoxins that are detrimental to the health of humans and animals. The *Alternaria* mycotoxins mainly include alternariol (AOH), alternariol monomethyl ether (AME) and tenuazonic acid (TeA) [2]. They frequently contaminate a variety of fruits, including tomatoes, apples, blueberries, grapes, and dried fruits, under suitable temperature and humidity conditions [1,3]. Of these mycotoxins, AOH and AME have been reported to show genotoxicity [4,5]. The genetic basis of AOH and AME biosynthesis has been elucidated and unraveled [6]. A polyketide synthase encoded by *pksI* is responsible and sufficient for AOH formation and an O-methyl transferase (omtI) transforms AOH to AME, the methyl ether of AOH. A pathway specific regulatory gene, *aohR*, encoding a Zn(II)2Cys6 transcription factor activated the expression of the downstream enzymatic genes for AOH and AME production.

In consideration of pathogenic *Alternaria* and the mycotoxins, the prevention and control is therefore of vital point. A number of plant extracts are considered to exert brilliant antifungal activities, including some naturally occurring polyphenols. Among the polyphenols, magnolol, extracted from the traditional Chinese herb *Magnolia officinalis*, showed strong antifungal activities against *Fusarium* species [7], *Candida* isolates [8], *Magnaporthe grisea* [9], *Penicillium expansum* as well as *A. alternata* [10]. However, little is known on its inhibitory effects against *Alternaria* toxins, especially AOH and AME. Accordingly, the deep exploration of antifungal and antimycotoxigenic mechanisms in response to magnolol are also still unclear in *A. alternata*.

Therefore, the antifungal and antimycotoxigenic efficiency of magnolol was first observed to be validated in the present work. The potential mechanisms were to be investigated by comparative transcriptomic profiling between the control and magnolol-stressed *A. alternata*. Given that magnolol was considered safe by various food safety authorities over the past years [11], it would be a promising alternative for preservatives for the control of *Alternaria* pathogens and their mycotoxins.

## 2. Results

### 2.1. Antifungal Effects of Magnolol on A. alternata

**Figure 1:**
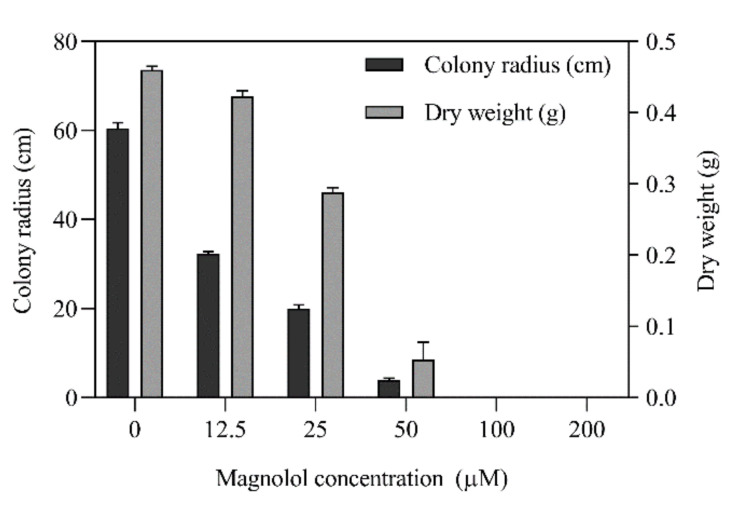
Inhibition of magnolol on the mycelial growth of *A. alternata* ATCC 66981. The spores were cultured in the medium plus with serial concentration of magnolol at 25 °C for 6 days. The inhibition rates were calculated by the colony radius and dry weight.

The antifungal effect of magnolol against *A. alternata* ATCC 66,981 was significantly exhibited on the mycelial growth in a dose-dependent manner. After 6 days incubation, the extension of *A. alternata* was inhibited by 46.5%, 66.9% and 93.5% with 12.5, 25 and 50 μM of magnolol, respectively (Figure 1). Similarly, the accumulation of the biomass weight was inhibited from 8.1% to 100% with the increasing magnolol (Figure 1). The extending of *A. alternata* was completely inhibited as the minimal inhibitory concentration (MIC) at 100 μM during the whole incubation period.

**Figure 2:**
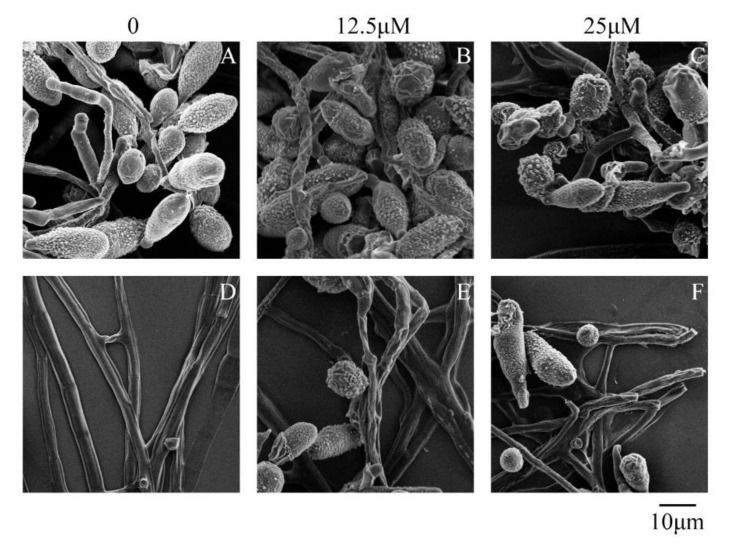
Scanning electron microscopy images of spores (**A**–**C**) and mycelia (**D**–**F**) of *A. alternata* ATCC 66,981 exposed to 0 (control), 12.5 and 25.0 μM of magnolol. Bar = 10 μm.

Ultrastructural observation by SEM indicated that there were significant morphological alterations between the control and magnolol-treated *A. alternata* cells. After exposure to 12.5 μM of magnolol, the spores were a little shriveled (Figure 2A,B). A greater depression of morphological modifications could be observed as the magnolol concentration increased. The exposure to 25 μM of magnolol caused an evidently sunken phenomenon and even a major break on the surface of the spore with no germ tubes observed (Figure 2C). The hyphae of *A. alternata* became wrinkled and abnormal after exposure to 12.5 and 25 μM of magnolol (Figure 2D–F).

### 2.2. Transcriptomic Profiles of A. alternata in Response to Magnolol

To understand the potential inhibitory mechanism of magnolol on *A. alternata*, a comparative transcriptome analysis of *A. alternata* was performed between 0 and 25 μM of magnolol treatment. The details of the RNA-Seq data are shown in Supplementary Table S1. An average of 37.00 million and 28.63 million clean reads were separately obtained from the control and magnolol-treated group. Based on the mapped reads, the expression levels of 13,678 genes were totally quantified by the FPKM (fragments per kilobase of exon model per million mapped reads) value. The differentially expressed genes (DEGs) in accordance with the |log_2_ Fold Change| ≥ 1 and false discovery rate (FDR) < 0.05 are listed in Supplementary Table S2. Totally, 2981 genes were considered to be significantly expressed in response to magnolol at 25 μM, with 1471 genes up-regulated and 1510 genes down-regulated compared to untreated *A. alternata* samples. The distribution of these DEGs is shown in Supplementary Figure S1.

### 2.3. Functional Analysis of DEGs

**Figure 3:**
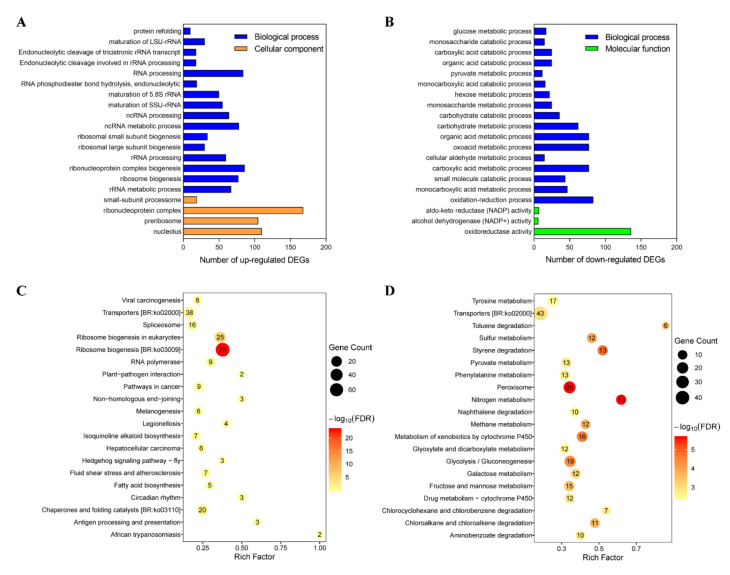
Gene ontology (GO) functional classification (**A**,**B**) and Kyoto Encyclopedia of Genes and Genomes (KEGG) pathway enrichment (**C**,**D**) of differentially expressed genes (DEGs). The up-regulated (**A**,**C**) and down-regulated (**B**,**D**) DEGs were separately analyzed and the top 20 results were listed at the highest enrichment level in the figure.

The functional analysis of DEGs was used to understand the inhibitory mechanisms at the molecular level. The significant results of GO enrichment analysis were apparently different between the up-regulated and down-regulated DEGs (Figure 3A,B). The up-regulated DEGs were significantly enriched in the processes of ribosome formation and RNA and protein processing; such as rRNA processing and metabolic processes, ribosome biogenesis, non-coding RNA (ncRNA) processing and metabolic processes, and RNA processing and protein refolding, which were necessary for the survival in exposure to stress. However, the down-regulated DEGs were mostly enriched in biological processes and molecular functioning, such as oxidoreductase activity, antibiotic metabolic processes and catabolic processes (Figure 3B).

KEGG pathway enrichment analysis of the DEGs between magnolol and magnolol-free groups was performed to evaluate their underlying biological significance. Genes related to the ribosome biogenesis showed significantly higher expression in the magnolol treated group (Figure 3C). On the other hand, the pathways mainly belonging to the peroxisome, nitrogen metabolism, and carbohydrate and energy metabolism were suppressed significantly in the magnolol stress samples (Figure 3D).

### 2.4. Genes Involved in Mycotoxin Biosynthesis

**Figure 4:**
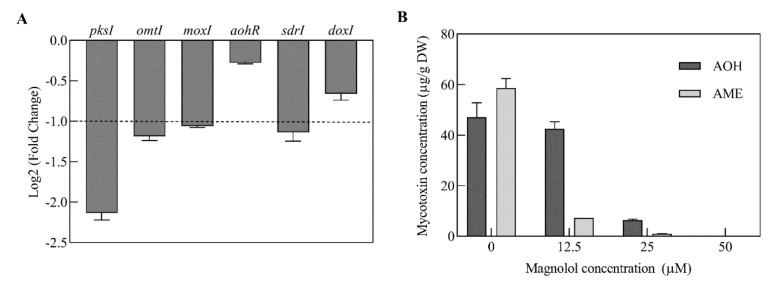
Effects of magnolol on the expressions of the clustered genes involved in mycotoxin biosynthesis (**A**), and alternariol (AOH) and alternariol monomethyl ether (AME) production (**B**) of *A. alternata* ATCC 66981. Each column represents the mean of three replicates and the results are presented as the mean ± standard errors (*p* < 0.05).

To understand the roles of magnolol on *Alternaria* mycotoxins production, the expression levels of genes involved in AOH and AME biosynthesis were analyzed. Based on the transcriptome data, the transcripts of *pksI* and *omtI*, responsible for AOH and AME biosynthesis, were significantly down-regulated by 4.39- and 2.28-fold when *A. alternata* was treated with 25 μM of magnolol, respectively, whereas the *aohR* showed no significant difference in expression (Figure 4A). The down-regulation of *pksI* and *omtI* may directly cause a reduction in AOH and AME biosynthesis. In addition, co-expression of *pksI* with another three enzymatic genes (*moxI, sdrI* and *doxI*) was also responsible for AOH modification [6]. Therein, the transcripts of two genes (*moxI* and *sdrI*) were significantly down-regulated in the 25 μM magnolol treated samples, while the *doxI* gene expression showed no significant differences (Figure 4A). The relative expression levels of three genes were down-regulated under magnolol stress, further confirming the results of transcriptomic analysis (Supplementary Figure S2). Meanwhile, AOH and AME production actually decreased dramatically by 86.2% and 98.2% in 25 μM of magnolol treated samples by the quantitative analysis (Figure 4B).

### 2.5. Genes Involved in the Primary Metabolism

Mycotoxin production by *A. alternata* is influenced by nutrition factors, such as carbon and nitrogen sources [12,13]. It is noteworthy that the influence of the nitrogen source for AOH and AME production is higher than that of the carbon source [12]. KEGG metabolic pathway analysis also showed that nitrogen metabolism was suppressed to a much stronger degree after magnolol treatment. The nitrogen metabolic regulators AreA and NmrA modulate gene expression for the utilization of nitrogen sources [14]. The expression of the global nitrogen metabolic regulator AreA exhibited no significant difference compared with the magnolol-free sample. However, the transcription of *nmrA* (*CC77DRAFT_1016967*) was significantly down-regulated by 2.90-fold in response to magnolol stress (Table 1). The nitrate assimilation system including nitrite reductase (NiR), nitrate reductase (NR) and a nitrate transporter NrtB was responsible for non-preferred nitrogen source utilization [15]. These genes showed lower expression from 4.31- to 5.19-fold by magnolol treatment (Table 1).

Carbon catabolite repression (CCR) plays an important role in the regulation of growth, development and secondary metabolism in mold [16,17]. As a principal regulator of CCR, CreA, mediates the carbon-utilizing systems. Previous studies have shown that the inactivation of *CreA* induced a marked decrease in aflatoxin biosynthesis [18,19]. In this work, the *CreA* (*CC77DRAFT_1058240*) expression level was significantly down-regulated by magnolol treatment, which suggests that CreA could also contribute to the reduction in AOH synthesis. In addition, CreA was regarded as a modulator of the cell wall integrity (CWI) pathway for the fungal resistance against antifungal agents [20], while RHO1—known as a small G protein—was considered the master regulator of CWI signaling [21]. Our data show that the presence of magnolol caused a significant down-expression of the RHO1 gene. Furthermore, some DEGs related to cell wall biogenesis markedly fluctuated by exposure to magnolol. Of the 11 DEGs, seven DEGs were down-regulated from 2.18- to 3.22-fold, and the other four DEGs were up-regulated from 2.37- to 5.82-fold (Table 1).

On the other hand, fatty acid β-oxidation contributes acetyl-CoA as a fundamental substrate for the polyketide mycotoxin production [19,22]. As shown in Table 1, all of the DEGs related to fatty acid β-oxidation were significantly less transcribed by 3.12- to 8.17-fold due to magnolol treatment. Fatty acid β-oxidation is carried out through the action of enzymes such as acyl-CoA dehydrogenase and 3-ketoacyl-CoA thiolase [23]. The expression levels of their encoding genes were down-regulated by 4.50- and 4.88-fold, respectively.

### 2.6. Genes Related to Stress Response

**Figure 5:**
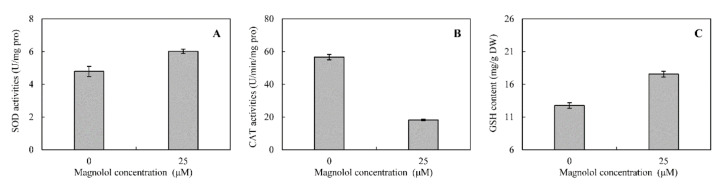
Effects of magnolol on the superoxide dismutase (SOD) and catalase (CAT) activities (**A**,**B**), and glutathione (GSH) content (**C**) of *A. alternata* ATCC 66981. SOD catalyzes the dismutation of superoxide radicals (O_2_^−^) to molecular oxygen (O_2_) and hydrogen peroxide (H_2_O_2_). CAT catalyzes the conversion of H_2_O_2_ to water and molecular oxygen. GSH is important as a cofactor for antioxidant enzymes, as a scavenger of ROS, and as a reducing agent for glutaredoxin. Each column represents the mean of three replicates. Results are presented as the mean ± standard error (*p* < 0.05).

To overcome oxidative stress, both enzymatic and non-enzymatic systems involving peroxidase (POD), superoxide dismutase (SOD), catalase (CAT) and glutathione (GSH) have been developed for scavenging the toxic ROS in fungi. SOD catalyzes the dismutation of superoxide radicals (O_2_^−^) to molecular oxygen (O_2_) and hydrogen peroxide (H_2_O_2_). CAT catalyzes the conversion of H_2_O_2_ to water and molecular oxygen. GSH is important as a cofactor for antioxidant enzymes, as a scavenger of ROS, and as a reducing agent for glutaredoxin. In this work, some DEGs involved in POD were up-regulated from 2.28- to 27.25-fold. Additionally, the expressions of two genes encoding SOD isozymes were also increased by 2.94- and 5.16-fold, respectively. However, the CAT encoding genes were all significantly down-regulated from 2.80- to 5.65-fold under the magnolol stress condition (Table 2). This was further confirmed by the assays of enzymatic activities, in which SOD activity in treated mycelia was enhanced by 25.6% (Figure 5A), whereas CAT activity was decreased by 41.4% compared to the control (Figure 5B).

Glutathione is the principal non-enzymatic compound protecting cells from oxidative stress [24]. As shown in Table 2, the DEGs related to GSH metabolism were down-regulated from KEGG analysis. Some glutathione S-transferases, which could catalyze the conjugation between GSH and many xenobiotic compounds for the purpose of detoxification, were less expressed by 2.72- and 8.89-fold. Correspondingly, the GSH contents were reduced by 20.7% in the magnolol treatment, compared to the control (Figure 5C). Moreover, sulfur-containing defense compounds involved in sulfur metabolism, including GSH and various sulfur-rich proteins, are crucial for fungal resistance against abiotic stress [25,26]. Our results showed that the expressions of 12 DEGs related to sulfur metabolism were totally repressed by magnolol.

In *A. alternata*, several oxidative stress-related proteins, mainly including the redox-responsive Yap1, the high osmolarity glycerol 1 (Hog1) mitogen-activated protein (MAP) kinase, the Skn7 response regulator and the non-ribosomal peptide synthetase (NPS6), have demonstrated to be involved in ROS modulation [27]. The transcriptomic profile showed that the expression of *Hog1* (*CC77DRAFT_164856*) was significantly down regulated by magnolol treatment. Conversely, an increase of 5.19-fold in the expression of *NPS6* (*CC77DRAFT_1061171*) was observed. NSP6, catalyzing the biosynthesis of siderophores, may assist the fungus to chelate iron and play a critical role for antioxidant activities and ROS detoxification [28]. Of the eight DEGs related to siderophore biosynthesis, except the major facility superfamily (MFS) transporter encoding gene, seven DEGs were overexpressed from 2.76- to 25.97-fold in response to the magnolol stress (Table 2). It is worth noting that the transcription factors Yap1 and Skn7 were demonstrated as having no significant change compared to the control.

## 3. Discussion

Magnolol is known as one of major bioactive compounds from *Magnolia officinalis* and has been reported to have biological functions such as anti-inflammatory, antifungal, anti-cancer and antioxidant activities [9,10]. Previous studies have showed that even at quite low concentrations magnolol exhibited high efficacy and strong potency against a broad spectrum of toxigenic fungi, including *Alternaria* spp. [9,10] and *Fusarium* spp. [7,9,29]. The mycelial extending and biomass weight of *A. alternata* in our work could be strongly inhibited by up to 93.5% and 88.3% with the treatment of magnolol only at 25 μM. Moreover, the MIC value of magnolol on *A. alternata* was much lower than that of other natural phenolics including *p*-coumaric, ferulic and caffeic acids [30,31]. Therefore, magnolol could be a potential alternative for controlling *Alternaria* decay. Chemical fungicides could possibly lead to fungal resistance and environmental problems. For this reason, many countries have established legislation to restrict the overuse of the chemical agents. The plant-derived magnolol is promising because of its non-phytotoxic, readily biodegradable, and environmentally safe properties. The major limitation of magnolol lies in its poor water solubility. Magnolol could be developed to be emulsified for antifungal efficiency. Additionally, the nanoparticle formulation of magnolol is recognized to be worthy to be explored for the improvement of its water solubility, such as the therapeutic formulation of magnolol nanoparticles [32].

Besides the pathogenic *Alternaria* fruits and their products, it is necessary to evaluate the magnolol effects on mycotoxins because little has been revealed. Of these mycotoxins, AOH and AME were demonstrated to be two of the most frequently contaminated mycotoxins [26,33]. To our knowledge, magnolol was first reported to have intense inhibitory effects on AOH and AME production in the present work, and 50 µM of magnolol could completely suppress mycotoxin biosynthesis. Similarly, type B trichothecene production by *F. culmorum* was significantly inhibited by magnolol treatment probably due to the presence of the free phenolic hydroxyl group on magnolol [29]. Actually, our previous studies showed that phenolics could be potent inhibitors against mycotoxin production in *A. alternata* [31]. Moreover, phenolics showed strong inhibition of aflatoxin and ochratoxin A biosynthesis by *Aspergillus* [19,34] and trichothecenes production by *Fusarium* [29,35]. Based on these results, the antifungal and antimycotoxigenic mechanism of magnolol on *A. alternata* growth and mycotoxin production was further investigated by transcriptomic analysis.

In *A. alternata*, the biosynthesis of AOH and its derivatives is managed by a polyketide gene cluster in which *aohR* serves as a positive transcriptional factor for mycotoxin production [6]. Our RNA-Seq data have shown that the clustered enzyme genes involved in AOH and AME biosynthesis were less transcribed, but for the expression of the regulatory gene *aohR* there was no significant decrease after the magnolol treatment. *PksI* was proven to be sufficient for AOH formation in *A. alternata*, while *omtI* was responsible for AME formation from the methyl ether of AOH [6]. Our previous study also found that the expression of *pksI* and *omtI* was strongly down-regulated by the essential oil citral, although the regulator *aohR* showed no significant change [26]. A similar result was reported by Zhao and his colleagues [19]. They found that most structural genes in the aflatoxin biosynthetic gene cluster were down-regulated while regulators *aflR* and *aflS* were not significantly affected by gallic acid [19]. Taken together, these finding indicated that magnolol inhibited mycotoxin production by lowering the expression of *pksI* and *omtI*.

Acetyl-CoA is the precursor for AOH formation [36], and it is mainly produced from fatty acid β-oxidation and pyruvate decarboxylation [19]. It was reported that gallic acid and cinnamaldehyde inhibited aflatoxin formation via the lower expression of fatty acid β-oxidation genes [19,37]. Our results also showed that all of the DEGs involved in fatty acid β-oxidation were highly repressed after exposure to magnolol. The last step of the β-oxidation cycle requires A 3-ketoacyl-CoA thiolase (KAT) enzyme which is essential for the last step of the β-oxidation cycle catalyzing fatty ketoacyl-CoA to produce one acetyl-CoA molecule [38]. It is worth noting that the expression of KAT was significantly down-regulated by 4.88-fold under magnolol stress. Therefore, the suppression of the DEGs responsible for the fatty acid β-oxidation pathway by magnolol would likely inhibit mycotoxin biosynthesis due to lack of precursors.

The growth and mycotoxin production in fungi is also influenced by the available carbon and nitrogen sources and the corresponding regulators [20,39,40]. The transcription factors CreA, AreA and NmrA play important roles in modulating the expression of genes for utilizing these nutrient sources [14,16,41]. Furthermore, CreA is essential for controlling secondary metabolism, either directly by interacting with the consensus sequences on the promoter region of mycotoxin biosynthetic genes or through transcriptional cascades and disruption of nutrient utilization [19]. Indeed, the deletion of *CreA* in *A. flavus* caused the significant repression of gene expression in aflatoxin biosynthesis and almost completely inhibited mycotoxin production [19,20]. Therefore, the downregulation of CreA could possibly lead to the decrease in AOH and AME production in *A. alternata* under the magnolol stress. In the nitrogen metabolic regulation, although there was no obvious significant difference in *AreA* expression, *NmrA* demonstrated lower levels of transcription in *A. alternata* with magnolol treatment. NmrA appears to affect gene expression through physical interaction with AreA [42], and also be involved in the mycotoxin biosynthesis and virulence of pathogens [18,40]. The *NmrA* deletion partially restricted the production of aflatoxin [40] and deoxynivalenol [43] in some minimal media with one amino acid as the sole nitrogen resource. These results suggest that the downregulation of *CreA* and *NmrA* under the magnolol stress may contribute to the significant reduction in AOH and AME production. It is noteworthy that CreA may be involved in nitrogen catabolite repression [41], while NmrA was considered to regulate carbon metabolism [44].

In addition to the nutritive factors, oxidative stress induced by environmental stimuli plays a crucial role in mycotoxin biosynthesis in fungi [24,45]. Oxidative stress was reported to stimulate the production of mycotoxins, including deoxynivalenol by *F. graminearum* [46], ochratoxin A by *A. ochraceus* [45] and aflatoxin by *A. flavus* [37] and *A. parasiticus* [47]. Likewise, fungi can cope with ROS toxicity by activating enzymatic and nonenzymatic defense systems, since excessive accumulation of ROS can lead to cellular dysfunction and even be lethal to cells. Several studies have reported that antioxidants could inhibit mycotoxin production by positive regulation of the antioxidant system and reducing the ROS in fungi [19,48]. However, a variety of inhibitors may act on the different types of antioxidant system. For example, gallic acid inhibited aflatoxin biosynthesis and stayed oxidative stress homeostasis in *A. flavus* by activating the nonenzymatic antioxidant system [19], while piperine depressed aflatoxin production accompanied with enhancement of CAT activity [48]. In this work, we found that exposure to magnolol led to the up-regulation of antioxidant enzymes in *A. alternata*, such as SOD and POD, while the CAT activity showed a sharp decrease. Similar trends were also observed by previous studies on the inhibitory effects of essential oils on the biosynthesis of patulin [49] and aflatoxin B_1_ [50]. They stated that essential oils suppressed mycotoxin production through enhancing SOD activity. In addition, siderophore-mediated iron uptake plays an important role in resistance to the toxic ROS which could be detoxified by SOD and CAT in *A. alternata* [28]. Our results show that almost all genes involved in siderophore biosynthesis were highly up-regulated after magnolol treatment. Meanwhile, iron present in excess could detoxify ROS via a non-enzymic system [28]. Taken together, it seems that the enhancement of SOD and POD activities and siderophore biosynthesis in *A. alternata* play important roles in the elimination of ROS and the inhibition of mycotoxin production by magnolol stress.

Oxidative stress leads to the response of gene transcription regulated by specific transcription factors and protein phosphorylation signaling pathways in fungi [24]. However, transcription factors like Yap and SKN7, which are responsible for regulating the oxidative response and ROS detoxification in *A. alternata* [27], did not show any significant difference under the magnolol condition. The other type is reported to be the modulation of the mitogen-activated protein kinase (MAPK) pathways [24]. Two MAPK pathways, the HOG and CWI pathways, are so vital for the adaptation of the cell to stress [51]. Our results showed that Hog1, the key MAPK of the HOG pathway, was significantly downregulated by magnolol. In fact, the Hog deletion mutant of *A. alternata* was almost unable to produce AOH, indicating that AOH biosynthesis required a phosphorylated HOG [52]. Additionally, Hog1 also modulates the production of several mycotoxins, such as fumonisin B_1_ [53] and ochratoxin A [54]. The CWI pathway is responsible for the maintenance of the cell wall, which is a common target for antifungal compounds [55,56]. This pathway is regulated by the RHO family of GTPases, of which RHO1 is considered the center regulator [21]. Previous studies have demonstrated that an increase in the transcription levels of RHO1 corresponded to a higher accumulation of AOH and AME, suggesting that the mycotoxin biosynthesis might be related to the CWI pathway [21,55]. Indeed, a significant inhibition of the RHO1 expression exposure to magnolol treatment was observed, indicating that the fungus was unable to activate the CWI pathway to overcome the stress. Meanwhile, the results from SEM microscopy also showed that magnolol destroyed cell wall integrity and permeability, which was consistent with the previous SEM observations on *A. alternata* treated with ethyl *p*-coumarate [30]. Taking the above into account, magnolol displayed strong antifungal and antimycotoxigenic activities against *A. alternata* by repressing the HOG and CWI signaling pathways and affecting cell wall integrity.

## 4. Conclusions

**Figure 6:**
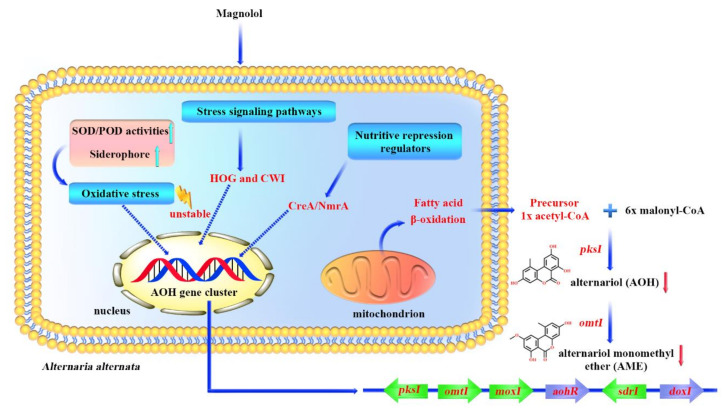
The schematic diagram demonstrating the inhibitory effects of magnolol against *A. alternata* and its mycotoxins. HOG: high osmolarity glycerol signaling pathway; CWI: cell wall integrity signaling pathway.

Magnolol exerts strong inhibitory effects on the growth and mycotoxin biosynthesis of *A. alternata* in this study, and the results provide a new insight into the global transcriptional alterations of *A. alternata* in response to magnolol stress. Based on the transcriptional profile, our results demonstrate in Figure 6 that: (1) magnolol inhibits the AOH and AME biosynthesis by down-expression of the polyketide clustered genes, including *pksI* and *omtI*; (2) the suppression of fatty acid β-oxidation after magnolol treatment leads to the marked reduction in mycotoxin precursor acetyl-CoA; (3) the down-regulation of nutritive repression regulators CreA and NmrA in response to magnolol may contribute to the significant decline in AOH and AME production; (4) magnolol enhances the expression of genes encoding antioxidant defense including SOD, POD and siderophore biosynthesis, together with the inhibition of the HOG and CWI signaling pathways, suggesting that magnolol inhibits AOH and AME production of *A. alternata* via the perturbation of the oxidative stress balance. In summary, magnolol could be a potential alternative to the traditional fungicides for controlling *Alternaria* decay and reducing mycotoxin contamination.

## 5. Materials and Methods

### 5.1. Strain and Culture Condition

*A. alternata* ATCC 66,981 was grown on potato dextrose agar (PDA; Becton Dickinson, Franklin Lakes, NJ, USA) at 25 °C for 7 days in order for the preparation of spore suspensions as the previous study [57]. The spore concentration was finally adjusted to 1 × 10^5^ spores/mL using a hemocytometer for the following assays.

### 5.2. Antifungal Effects of Magnolol on A. alternata

Magnolol, purchased from Sigma-Aldrich (St. Louis, MO, USA), was dissolved with 50% aqueous ethanol (*v*/*v*) to prepare for the stock solution. It was mixed with the medium to obtain the different concentrations (0, 12.5, 25, 50, 100 and 200 μM). Antifungal trials of magnolol were performed as our previous work with slight modifications [31]. A total of 5 μL aliquot of spore suspension was inoculated on PDA medium with serial concentrations of magnolol and cultured at 25 °C for 6 days for diameter determination. In addition, liquid culture was carried out using potato dextrose broth (PDB; Becton Dickinson, Franklin Lakes, NJ, USA) at the final concentration of 10^2^ spores/mL with shake flask culture in an incubator at 25 °C, 180 r/min. The minimum inhibitory concentration (MIC) was recognized as the lowest concentration of magnolol without visible fungal extending. After incubation for 6 d, the culture was filtered with Whatman filter paper. The supernatant was used for the determination of AOH and AME contents, while the mycelium was freeze-dried for the biomass analysis or quickly frozen with liquid nitrogen for the following RNA extraction. Each test was performed in triplicate. The inhibition ratio of the mycelial growth and biomass weight was separately calculated.

### 5.3. Scanning Electron Microscopy (SEM) Analysis

The mycelia of *A. alternata* were collected and washed three times using phosphate buffered saline (PBS; pH 7.4) after 72 h incubation. The mycelia were mixed with PDB medium with 0, 12.5 or 25 μM of magnolol (mycelial weight: the volume of the medium = 1:10).

For the analysis of spore morphological changes, spore suspension was mixed with the antifungal agent to get the magnolol final concentrations of 0, 12.5 and 25 μM. After incubation at 25 °C, 180 r/min for 12 h, both mycelia and spores were centrifuged for 5 min at 10,000× *g*, resuspended with PBS to remove the residue of medium and magnolol. The samples were then followed by serial pretreatments for the microscope observation based on Wang et al. [26]. Finally, the morphological alteration was compared using a scanning electron microscope (S-3400N, Hitachi, Tokyo, Japan).

### 5.4. AOH and AME Analysis

The extraction of AOH and AME was carried out following by the method of Meena et al. [58] with minor modification. The culture was added with equal volume of acetonitrile containing 0.1% (*v*/*v*) formic acid, vortexed for 3 min and then kept at 4 °C for 24 h. After the centrifugation, the supernatant was processed and AOH and AME accumulation was quantified under the direction of Wang et al. [26].

### 5.5. RNA Extraction, cDNA Library Construction, and RNA-Seq Analysis

RNA was extracted from 6 days cultured mycelia from *A. alternata* ATCC 66981 grown in PDB medium with or without 25 μM of magnolol using TRIzol reagent (Invitrogen, Carlsbad, CA, USA) following the manufacturer’s instructions. Each group has three biological replications. Extracted RNA was evaluated with the agarose gel electrophoresis and the Qubit RNA Assay Kit (Invitrogen, Carlsbad, CA, USA). The cDNA libraries construction and RNA-seq was performed at Beijing Geek Gene Technology Co., Ltd. (Beijing, China).

The reads adapters, low quality reads, and reads with more than 5% N were to be trimmed from the raw reads. The remaining reads were conducted to be bioinformatically analyzed based on the genome annotation of *A. alternata* (genome assembly: GCF_001642055.1) [27]. The genes of significantly differential expression between these compared samples were analyzed. Finally, gene ontology (GO) functional annotation and Kyoto Encyclopedia of Genes and Genomes (KEGG) pathway enrichment analysis were carried out to understand the functions of the differentially expressed genes (DEGs). To further confirm the reliability of the results from the gene expression analysis by RNA-Seq, quantitative reverse transcription polymerase chain reaction (qRT-PCR) was conducted for the clustered genes that were involved in AOH and AME biosynthesis by the reference of Wang et al. [26].

### 5.6. Antioxidant Enzymatic Activities and Glutathione Measurement

To evaluate the impact of magnolol on the antioxidant system activities in *A. alternata,* the analyses of superoxide dismutase (SOD), catalase (CAT) and glutathione (GSH) were performed following the same culture conditions of RNA extraction. The enzymatic activities of SOD and CAT, and the content of GSH were determined by the respective detection kits (Solarbio, Beijing, China) following the instructions.

### 5.7. Statistical Analysis

Statistical analyses of all the results were completed by Microsoft Excel 2016. The significant difference of each treatment was compared by one-way analysis of variance (ANOVA) with the least significant differences’ test (LSD test) at *p* < 0.05 using IBM SPSS Statistics 21.0 (SPSS Inc., Chicago, IL, USA).

## Figures and Tables

**Table 1:** Effects of magnolol treatment on the differential expression of genes related to the primary metabolites in *A. alternata*.

Gene	Log_2_ (AaM/AaC) ^1^	*p*-Value	Function
Nitrogen metabolism			
*CC77DRAFT_1016967*	−1.536	0.001	nmrA
*CC77DRAFT_1034395*	−2.647	0.000	2-nitropropane dioxygenase precursor
*CC77DRAFT_1056077*	−2.376	0.013	high affinity nitrate transporter NrtB
*CC77DRAFT_937904*	−2.108	0.004	nitrite reductase NiR
*CC77DRAFT_937944*	−2.159	0.001	nitrate reductase NR
*CC77DRAFT_1011175*	−2.026	0.000	cyanate hydratase
*CC77DRAFT_383282*	−1.938	0.000	formamidase FmdS
*CC77DRAFT_937213*	−1.831	0.000	carbonic anhydrase
*CC77DRAFT_854456*	−1.897	0.004	putative oxidoreductase
*CC77DRAFT_201495*	−1.541	0.000	carbon-nitrogen hydrolase
*CC77DRAFT_1059060*	−1.677	0.000	carbonic anhydrase
*CC77DRAFT_505636*	−1.650	0.000	glutamine synthetase
*CC77DRAFT_261749*	−1.627	0.000	glutamate synthase
*CC77DRAFT_926595*	−1.065	0.000	glutamine synthetase
Carbon utilization			
*CC77DRAFT_1058240*	−1.289	0.000	CreA
*CC77DRAFT_318215*	−2.128	0.000	C_2_H_2_ transcription factor (AmdA)
*CC77DRAFT_947345*	−1.766	0.000	acetyl-CoA synthetase-like protein
*CC77DRAFT_1063927*	−1.715	0.000	C6 transcription factor FacB
*CC77DRAFT_1020016*	−1.526	0.000	pyruvate carboxylase
*CC77DRAFT_30145*	−1.177	0.000	mitochondrial uncoupling protein 2
*CC77DRAFT_997221*	−1.015	0.000	mitochondrial carrier
*CC77DRAFT_926519*	1.412	0.000	isocitrate lyase 2
Cell wall biogenesis			
*CC77DRAFT_598626*	−1.007	0.000	Rho GTPase1
*CC77DRAFT_1022845*	−1.230	0.000	mannose-6-phosphate isomerase
*CC77DRAFT_1066075*	−1.526	0.000	mannose-6-phosphate isomerase
*CC77DRAFT_944335*	−1.685	0.000	quinone oxidoreductase putative
*CC77DRAFT_61983*	−1.211	0.000	chitinase
*CC77DRAFT_985840*	−1.195	0.007	chitin deacetylase 1
*CC77DRAFT_2778*	1.515	0.000	chitinase
*CC77DRAFT_1061411*	1.247	0.000	nucleotide-diphospho-sugar transferase
*CC77DRAFT_1039824*	1.335	0.000	hypothetical protein
*CC77DRAFT_1064369*	2.536	0.000	glycoside hydrolase
*CC77DRAFT_906789*	−1.612	0.009	glycoside hydrolase
*CC77DRAFT_931389*	−1.127	0.004	glycoside hydrolase
Fatty acid beta-oxidation			
*CC77DRAFT_937137*	−3.031	0.000	tropinone reductase 1
*CC77DRAFT_1024610*	−2.286	0.000	3-ketoacyl-CoA thiolase B
*CC77DRAFT_38276*	−2.169	0.000	acyl-CoA dehydrogenase
*CC77DRAFT_1087261*	−1.963	0.021	Delta3-Delta2-enoyl-CoA isomerase
*CC77DRAFT_210867*	−1.642	0.000	3-oxoacyl-reductase

^1^ AaC, control; AaM, magnolol treatment. Log_2_ (AaM/AaC) ≥ 1 indicates up-regulated expression and Log_2_ (AaM/AaC) ≤ −1 indicates down-regulated expression.

**Table 2:** Effects of magnolol treatment on the significantly differential expression of the genes encoding for the antioxidant activities in *A. alternata*.

Gene	Log_2_ (AaM/AaC) ^1^	*p*-Value	Function
Antioxidant enzyme			
*CC77DRAFT_905830*	1.554	0.000	Superoxide dismutase (SOD)
*CC77DRAFT_911584*	2.368	0.000	SOD
*CC77DRAFT_1050117*	−1.644	0.000	SOD
*CC77DRAFT_1021907*	−1.374	0.000	SOD
*CC77DRAFT_1036489*	−2.497	0.001	Catalase (CAT)
*CC77DRAFT_1013212*	−1.485	0.000	CAT
*CC77DRAFT_296007*	3.497	0.000	Peroxidase (POD)
*CC77DRAFT_299044*	3.421	0.000	POD
*CC77DRAFT_1039208*	1.391	0.000	POD
*CC77DRAFT_960122*	1.186	0.000	POD
*CC77DRAFT_1021328*	4.768	0.000	POD
*CC77DRAFT_227615*	1.812	0.000	POD
*CC77DRAFT_1096817*	−2.209	0.001	POD
*CC77DRAFT_1051184*	−1.353	0.015	POD
*CC77DRAFT_1008747*	−1.162	0.000	POD
Glutathione metabolism			
*CC77DRAFT_1032529*	−3.152	0.000	glutathione S-transferase II
*CC77DRAFT_687848*	−1.445	0.000	glutathione S-transferase II
*CC77DRAFT_1050574*	−1.671	0.000	glutamate-cysteine ligase
*CC77DRAFT_1022906*	−1.210	0.000	glutamate-cysteine ligase regulatory subunit
*CC77DRAFT_77574*	−2.371	0.000	thioredoxin-like protein
*CC77DRAFT_1056344*	1.282	0.001	glutathione S-transferase
*CC77DRAFT_356794*	1.067	0.001	glutathione S-transferase
Siderophore biosynthesis			
*CC77DRAFT_935643*	1.464	0.000	tyrosinase
*CC77DRAFT_1061171*	2.376	0.000	Non-ribosomal peptide synthase, NPS6
*CC77DRAFT_1031046*	2.732	0.000	ABC transporter
*CC77DRAFT_1020276*	4.384	0.000	L-ornithine 5-monooxygenase
*CC77DRAFT_69570*	2.126	0.000	aerobactin siderophore biosynthesis protein iucB
*CC77DRAFT_69691*	4.699	0.000	short-chain-fatty-acid-CoA ligase, sidI
*CC77DRAFT_71629*	4.417	0.000	siderophore iron transporter, mirB
*CC77DRAFT_69581*	−2.210	0.000	MFS transporter
Sulfur metabolism			
*CC77DRAFT_1015694*	−3.069	0.000	thiosulfate sulfurtransferas-like protein
*CC77DRAFT_1020633*	−1.427	0.000	sulfite reductase hemoprotein
*CC77DRAFT_1022955*	−3.093	0.000	o-acetylhomoserine ami
*CC77DRAFT_1067622*	−2.515	0.000	molybdopterin binding oxidoreductase
*CC77DRAFT_1099663*	−1.502	0.000	sulfide: quinone oxidoreductase
*CC77DRAFT_675919*	−1.094	0.000	carbohydrate phosphatase
*CC77DRAFT_786633*	−1.703	0.000	adenylyl-sulfate kinase
*CC77DRAFT_925110*	−2.268	0.000	phosphoadenosine phosphosulfate reductase
*CC77DRAFT_951586*	−1.374	0.000	ATP-sulfurylase
*CC77DRAFT_971236*	−5.474	0.000	methanesulfonate monooxygenase
*CC77DRAFT_192219*	−1.215	0.000	alpha/beta-hydrolase
*CC77DRAFT_240786*	−1.437	0.000	homoserine acetyltransferase family protein

^1^ AaC, control; AaM, magnolol treatment. Log_2_ (AaM/AaC) ≥ 1 indicates up-regulated expression and Log_2_ (AaM/AaC) ≤ −1 indicates down-regulated expression.

## Supplementary Materials

The following are available online at https://www.mdpi.com/2072-6651/12/10/665/s1. Figure S1: Volcano plot of these differentially expressed genes (DEGs) of *A. alternata* treated with 25 μM of magnolol vs. control, Figure S2: Comparative analysis of gene expression of *A. alternata* in 0, 12.5, 25 μM of magnolol by quantitative reverse transcription PCR (qRT-PCR), Table S1: Summary of the RNA-Seq data in the control (AaC) and 25.0 μM magnolol treated (AaM) groups of *A. alternata* ATCC 66981, Table S2: Differentially expressed genes (DEGs) between the control and magnolol stressed samples of *A. alternata*.
