# Supplementary material for: Transcriptomic Insights into the Antifungal Effects of Magnolol on the Growth and Mycotoxin Production of Alternaria alternata
Source: Toxins (Basel). 2020 Oct 20;12(10):665. doi: 10.3390/toxins12100665 (PMC7594048; doi:10.3390/toxins12100665)
Supplement: Supplementary file 1 [file toxins-12-00665-s001.zip › toxins-912667 supplementary final/toxins-912667 supplementary.pdf]

Supplementary Materials: Transcriptomic Insights into the Antifungal Effects of Magnolol on the Growth and Mycotoxin Production of *Alternaria alternata*

Liuqing Wang, Duo Wang, Shuzhi Yuan, Xiaoyuan Feng and Meng Wang

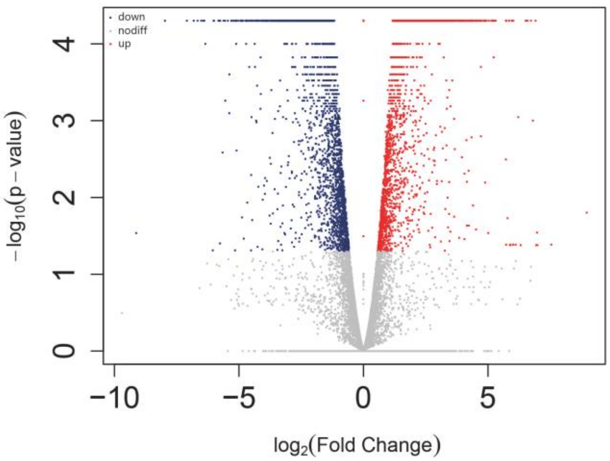

Figure S1. Volcano plot of these differentially expressed genes (DEGs) of *A. alternata* treated with 25 μM of magnolol vs. control.

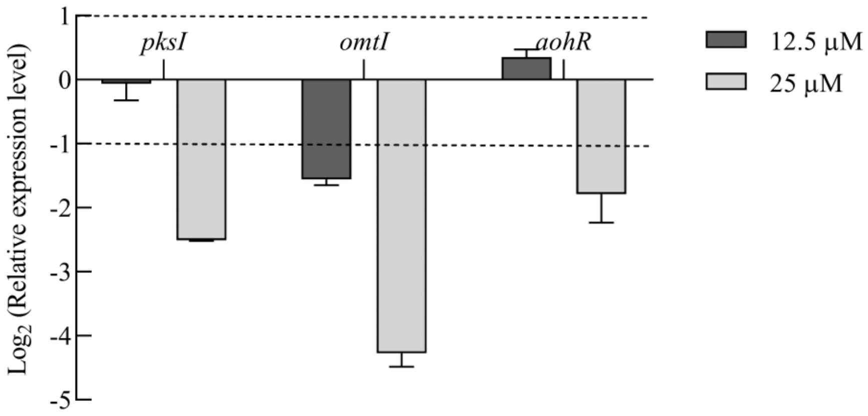

Figure S2. Comparative analysis of gene expression of *A. alternata* in 0, 12.5, 25 μM of magnolol by quantitative reverse transcription PCR (qRT-PCR). Genes of *pksI*, *omtI* and *aohR* were directly responsible for AOH and AME biosynthesis and regulation in *A. alternata*.

Table S1. Summary of the RNA-Seq data in the control (AaC) and 25.0 μM magnolol treated (AaM) groups of *A. alternata* ATCC 66981.

Parameter	AaC1	AaC2	AaC3	AaM1	AaM2	AaM3
Raw Reads	43,177,416	39,142,004	32,894,838	30,449,858	25,426,692	36,605,620
Clean Reads	41,119,788	37,963,424	31,906,964	28,276,536	23,238,714	34,370,630
Clean Reads Rate (%)	95.23	96.99	97.00	92.86	91.39	93.89
Mapped Reads	23,697,435	21,809,297	19,023,683	15,885,293	12,617,501	20,203,599
Mapping Rate (%)	57.63	57.45	59.62	56.18	54.30	58.78
MultiMap Reads	95,684	80,040	89,944	46,424	36,212	73,576
MultiMap Rate (%)	0.40	0.37	0.47	0.29	0.29	0.36
Q20 (%)	94.80	95.21	95.20	93.53	92.85	93.87
Q30 (%)	90.21	90.50	90.22	88.63	87.86	88.72
GC content (%)	52.93	53.33	53.13	52.69	52.74	53.27
